# Penetrating abdominal injury and peritonitis: A rare case of birth Injury

**DOI:** 10.4103/0971-9261.42569

**Published:** 2008

**Authors:** Shreeprasad P. Patankar, Shilpa S. Patankar

**Affiliations:** Department of Pediatric Surgery, Bharati Vidyapeeth University's Bharati Hospital and Medical College, Dhanakawadi, Satara Road, Pune, Maharashatra, India

**Keywords:** Birth injury, bowel trauma, penetrating abdominal trauma

## Abstract

The incidence of birth injuries has decreased considerably because of the identification of risk factors at an earlier stage and taking the decision for caesarian section (LSCS) at proper time. Fractures, nerve palsies and central nervous system injuries comprise the majority of “birth injuries.” In this study, we report a newborn that had a birth injury during LSCS. The baby sustained a penetrating abdominal injury by the knife of the surgeon, while performing LSCS. The bowel was injured at two sites, proximal jejunum and descending colon. The baby developed meconeum spillage and peritonitis. Exploratory laprotomy was done and the injuries were identified. The injured portions were resected and bowel continuity was reestablished. The baby had an uneventful recovery.

Injuries to the newborn as a result of traction, mechanical forces during birth process are called “Birth Injuries.” There are some risk factors involved that predispose the baby to such injuries. The incidence of birth injuries is 6-8 cases in 1000 live births. The common types of birth injuries are CNS trauma, extracranial or intracranial hematoma, nerve palsies, fractures of limb bones and hematoma in solid abdominal organs. With the liberal use of Caesarian deliveries, the “pull-and-traction”-induced injuries are reducing in number; however, we may still encounter “cut-and-incise”-type of injuries. We present here a rare case of birth injury where the intestines of an infant got traumatized with the knife of the surgeon during the Caesarian section.

## CASE REPORT

A full-term male newborn weighing 2500 g was born with LSCS. The indication of LSCS was oligohydramnios, breech presentation and fetal distress. While performing the LSCS, the incision on the lower segment of the uterus resulted into a full-thickness muscle-cutting incised wound in the left subcostal region and left flank of the baby. The intestinal coils were eviscerated and there was trauma to the bowel, which was evident by spillage of meconeum. The operating doctor identified the colonic injury and sutured the colon with 3/0 silk in two layers. He reposed the intestinal coils into the abdomen of the baby and closed the abdomen. The baby was shifted to NICU for further management. He was managed with IV fluids, antibiotics and continuous nasogastric tube aspiration. The baby did not pass meconeum, had progressive abdominal distension and had repeated bilious vomiting. It was the sixth postoperative day, when it was referred to us for further management. The baby had lost 300 g of weight; the vital parameters were within normal limits. Abdomen looked distended with prominent intestinal loops. The abdominal wall was tense, shiny, with dilated veins and periumbilical erythema. There was a sutured 4-cm-long incised wound extending from the left sub-scapular region, going across the distal ends of eleventh and twelfth ribs that reached up to the left flank area. There was bulge in the left flank and lateral portion of abdomen whenever baby cried, indicating damage in the left subcostal nerve. Erect abdominal X-ray showed dilated bowel loops and multiple air fluid levels. Hemogram, serum electrolytes and renal function tests were normal.

**Figure 1:**
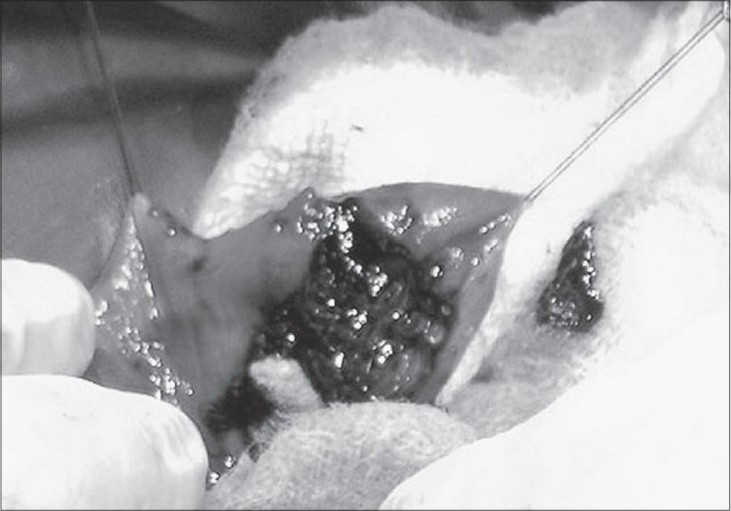
Injury on antimesenteric side of jejunum with meconeum spillage

**Figure 2:**
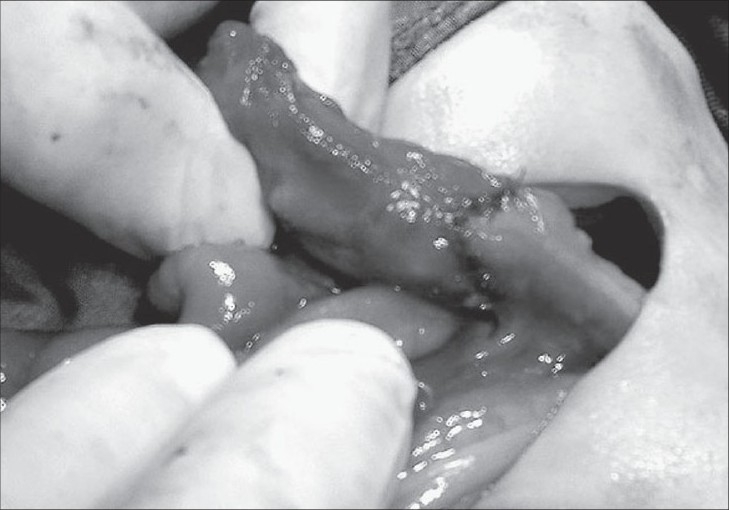
Injured and sutured portion of the colon that is resected and after anastomosis

The infant was taken for exploratory laprotomy on seventh day of life. Left supraumbilical transverse incision was taken; ascitis was evident. The small intestinal coils were distended with air and meconeum. There were inflammatory adhesions between the small intestinal coils in the region of proximal jejunum, descending mesocolon and descending colon. The adhesions were carefully separated. There was a perforation on the antimesenteric wall of the jejunum that was approximately 2 cm distal to duodenojejunal flexure [Figure 1]. There was meconeum spillage from the above mentioned perforation. The point of obstruction was found to be the descending colon that was injured and sutured with silk sutures. The other viscera and spleen were normal. A segment of descending colon (measuring 2 cm) that involved the injured-sutured portion was excised and oblique end-to-end anastomosis was done with 5/0 vicryl [Figure 2]. The edges of the jejunal perforation were freshened and enterotomy wound was closed transversely with 5/0 vicryl. Thorough peritoneal lavage was given, and the abdomen was closed after maintaining drain. The baby recovered well and was discharged on the fifteenth postoperative day. On follow up, the baby thrived well.

## DISCUSSION

The process of childbirth involves a very intricate interplay of propulsive force generated by the uterine musculature and liquor, molding of silhouette of the infant and expansion of the birth canal. Abnormal fetal presentation, extremes of fetal size or neurologic immaturity may complicate the birth process and the intrapartum forces may lead to tissue damage, edema, hemorrhage or fracture in the neonate. Such injuries to the infant resulting from mechanical forces (i.e., compression and traction) during the birth process are categorized as birth trauma.[1–3] It still occurs occasionally and unavoidably, with an average of 6-8 injuries per 1000 live births.[2] The use of obstetric instrumentation may add to the list of forces or may induce injury alone. Risk factors include large-for-date infants, particularly infants who are weigh more than 4500 g; instrumental deliveries, especially forceps (midcavity) or vacuum; vaginal breech delivery; precipitous labor; various obstetric version maneuvers and abnormal or excessive traction during delivery.[1–3]

The majority of birth injuries are potentially avoidable with recognition and anticipation of obstetric risk factors. Under certain conditions, delivery by cesarean section can be an acceptable alternative, but it does not guarantee an injury-free birth. It has given rise to a new variety of birth trauma: “trauma caused by surgeon's knife.” Small linear incised wounds are known to occur occasionally and are taken care by primary suturing. However, deep incision that has cut along all the abdominal muscle layers and injury to bowel coils is extremely rare event.

This case is a unique example of such a rare event. Multiple risk factors, i.e., oligohydramnios and breech presentation of the baby, seem to be present in this case acting together to create a situation to precipitate the injury.

Abnormal presentation has brought the flank in close approximation with the anterior uterine wall. The flexion attitude of the silhouette of the infant might have stretched the body wall in the flank of the infant. The absence of sufficient amniotic fluid obscured the anatomical differentiation between uterine wall and body wall of the infant. Hence the knife of the surgeon traumatized the abdominal wall of the infant as soon as it entered the uterine cavity. The knife entered from the left flank and traumatized the bowel in the vicinity and the descending colon, which is a retroperitoneal organ in left flank. The duodenojejunal flexure and proximal jejunum is also located in left paraspinal region; hence, it was traumatized. It is surprising to note that left kidney and the upper portion of left ureter that lies in the same region were spared from injury.

Intraabdominal birth injuries are rare and involve rupture or subcapsular hemorrhage into the liver, spleen or adrenal gland. Most of these injuries are self-limiting; however, they require close monitoring and follow up. Rarely, they can complicate in hemoperitoneum and may indicate surgical intervention.[1–6] This is rarest of rare cases where a penetrating abdominal birth injury has occurred and being treated successfully. We will like to add here that pediatric surgeon should have been involved as early as possible. That could have rendered an early thorough exploration, suturing of injured bowel and prevention of morbidity because of peritonitis.

These type of events also has serious medicolegal aspects. A newborn who has sustained birth injury is a source of great concern for the parents, obstetrician and pediatrician. Although treating the affected baby is the primary concern for the involved clinicians, such event may initiate litigation, particularly when the parents search the Internet to obtain information on the birth injury of the baby and come across a number of websites from malpractice lawyers, promising them legal action against the doctor.[3–6]
